# Sports Injuries and Mental Health in Child and Adolescent Athletes: A Systematic Review

**DOI:** 10.3390/children13081076

**Published:** 2026-08-14

**Authors:** Laura Gil-Caselles, Aurelio Olmedilla-Zafra

**Affiliations:** 1 Research Group HUMSE, Faculty of Sports Sciences, University of Murcia, 30720 Murcia, Spain; 2 Department of Personality, Psychological Assessment and Treatment, Faculty of Psychology, University of Murcia, 30100 Murcia, Spain; olmedilla@um.es

**Keywords:** psychological well-being, injury vulnerability, sleep disturbance, fear of reinjury, return-to-sport readiness

## Abstract

**Highlights:**

**What are the main findings?**

- Sports injuries and mental health appear to be bidirectionally associated in child and adolescent athletes.
- Psychological well-being, anxiety, stress, sleep, fatigue, fear of reinjury, and psychological readiness are relevant across injury occurrence, recovery, and return to sport.

**What are the implication of the main findings?**

- Injury management may benefit from integrating developmentally appropriate psychological assessment with physical rehabilitation.
- Psychological factors should be monitored throughout recovery and interpreted within the athlete’s broader clinical, developmental, and sporting context.

**Abstract:**

Background: Sports injuries in children and adolescents may affect physical health, emotional well-being, sleep, quality of life, confidence, sport participation, and readiness to return to sport. Objective: This systematic review synthesized evidence on the relationship between sports-related injuries and mental health in athletes aged 19 years or younger, considering both psychological factors associated with injury vulnerability and psychosocial outcomes following injury. Methods: The review was conducted in accordance with the PRISMA 2020 guidelines. PubMed, Web of Science, Scopus, and Google Scholar were searched from inception to 8 July 2026, with eligibility restricted to studies published between 2021 and 2026. Risk of bias was assessed using Joanna Briggs Institute critical appraisal tools, and findings were synthesized narratively. Results: Twenty-one studies were included. Lower well-being, anxiety, stress, fatigue, sleep disturbance, kinesiophobia, and reduced psychological readiness were associated with injury occurrence, symptom burden, recovery, or reinjury. Conversely, injuries were associated with anxiety, depressive symptoms, sleep problems, reduced health-related quality of life, lower confidence, disrupted athletic identity, and return-to-sport difficulties. Conclusions: The findings suggest a potentially bidirectional association between sports injury and mental health within a broader biopsychosocial context. However, heterogeneity and predominantly observational designs limit causal inference.

## 1. Introduction

Participation in organized sport during childhood and adolescence is widely recognized as an important contributor to physical, psychological, and social development. Sport provides opportunities to improve physical fitness, develop motor skills, strengthen peer relationships, promote emotional regulation, and establish healthy lifestyle habits. However, sports participation also exposes young athletes to acute, recurrent, and overuse injuries that may disrupt training, competition, academic routines, and social interaction [1,2,3,4]. In children and adolescents, the consequences of sport injury should not be interpreted exclusively from a physical or biomechanical perspective, as injury occurs during a sensitive developmental period in which identity, autonomy, self-esteem, emotional regulation, and coping strategies are still developing [1,2,5]. In this context, sports injury encompasses acute, recurrent, and overuse conditions resulting from sport participation that may restrict training, competition, or normal daily functioning [1,2,3,4].

The psychosocial impact of sports-related injuries in young athletes has received increasing attention in pediatric sports medicine. Injured children and adolescents may experience frustration, sadness, anger, isolation, reduced motivation, fear of reinjury, uncertainty about recovery, and loss of confidence during rehabilitation and return to sport [1,2,6,7,8,9]. These responses may be particularly relevant during adolescence, when sport participation often becomes closely linked to daily routines, social belonging, athletic identity, and future aspirations. Consequently, injury may represent not only a temporary physical limitation, but also a disruption of self-perception, peer connection, and perceived competence [5,10]. Moreover, psychological responses observed in adult athletes cannot necessarily be extrapolated to younger populations, because children and adolescents differ in emotional development, dependence on family and coaches, coping resources, and understanding of injury and recovery [1,2,5,9].

Recent evidence has directly addressed the bidirectional relationship between sports injury and mental health in young athletes. Chow et al. [11] conducted a preregistered systematic review and meta-analysis of 84 quantitative studies involving 221,095 adolescents and young adults aged 10–24 years. Their findings supported associations in both directions: sports injuries were associated with poorer mental health and well-being, whereas poorer mental health and well-being were associated with a higher risk of subsequent sports injury. However, the latter association was no longer statistically significant after adjustment for potential publication bias, underscoring the need for caution when interpreting psychological factors as predictors of injury.

Several psychological factors may influence the injury process, including competitive anxiety, depressive symptoms, fear of reinjury, pain catastrophizing, coping strategies, perceived stress, resilience, athletic identity, and social support [7,10,12,13,14]. Fear of reinjury is one of the most frequently reported psychological barriers after sport injury and may negatively affect rehabilitation adherence, movement confidence, performance, and psychological readiness to return to sport [14]. Similarly, athletic identity may intensify the emotional consequences of injury, as young athletes who define themselves strongly through sport may experience greater distress when they are unable to train or compete [13]. These findings support the importance of considering psychological recovery as an integral component of injury management in pediatric and adolescent sport.

The broader sport context may also shape the relationship between injury and mental health. Early sport specialization, excessive training volume, overtraining, and burnout have been associated with both physical and psychosocial risks in youth athletes [4,5]. Likewise, sport type and competitive environment may influence psychological outcomes, as team sports may provide protective social support, whereas individual sports may involve greater perceived pressure and individual responsibility for performance [6]. Therefore, injury-related mental health outcomes should be understood within a biopsychosocial framework that incorporates the athlete’s developmental stage, previous injury history, family support, coach communication, peer relationships, sport demands, and return-to-sport expectations [2,10,12,15]. Recent literature has further emphasized the mental health and psychological consequences of sport participation and injury in pediatric athletes [9], as well as the potential contribution of sport-psychology approaches to injury prevention and psychological risk management [16].

The present review is positioned as a complementary and updated synthesis rather than as the first review to examine bidirectionality. In contrast to Chow et al. [11], it applies a stricter upper age limit of 19 years, thereby excluding young adults aged 20–24 years, and incorporates evidence published through 8 July 2026. Of the 21 studies included in the present review, only two overlapped with the studies included by Chow et al. [11]; 13 were published between 2024 and 2026 and therefore fell outside their search period. Accordingly, the added contribution of this review lies primarily in providing an updated synthesis focused specifically on child and adolescent athletes aged 19 years or younger, covering psychological and psychosocial factors across injury occurrence, rehabilitation, and return to sport.

On this basis, the review was guided by the following question: how are sports-related injuries and mental health associated in child and adolescent athletes throughout injury occurrence, recovery, and return to sport? The main objective was therefore to synthesize the available evidence on this relationship. Specifically, the review aimed to identify mental health outcomes associated with sports injuries, examine whether psychological factors may act as risk factors or consequences of injury, and describe the main psychosocial variables involved in injury occurrence, rehabilitation, and return to sport in youth athletes.

## 2. Materials and Methods

### 2.1. Design

This systematic review was conducted and reported in accordance with the Preferred Reporting Items for Systematic Reviews and Meta-Analyses 2020 statement (PRISMA 2020) [17]. The review was designed to identify, evaluate, and synthesize the available evidence on the relationship between sports-related injuries and mental health outcomes in child and adolescent athletes. Bidirectionality was operationalized a priori by examining two complementary directions of association: psychological or psychosocial factors preceding or associated with subsequent sports injury, and psychological or psychosocial outcomes occurring following sports injury. The review process included the formulation of the research question, definition of eligibility criteria, systematic literature searching, study selection, data extraction, risk-of-bias assessment, and narrative synthesis of the findings. The review protocol was developed a priori and was subsequently registered retrospectively in PROSPERO (CRD420261459540).

### 2.2. Eligibility Criteria

Studies were included if they: (1) involved children or adolescents aged 19 years or younger who participated in organized or competitive sport; (2) examined a sports-related injury, injury history, injury risk, rehabilitation, or return to sport; (3) assessed at least one mental health or psychosocial variable in relation to injury; and (4) reported original empirical data.

Eligible study designs included observational studies, qualitative studies, case series, and secondary analyses of randomized or non-randomized intervention studies, provided that they met the predefined population, sports-injury, and psychological or psychosocial outcome criteria.

The upper age limit of 19 years was selected to encompass the full adolescent developmental period, consistent with the World Health Organization definition of adolescence as 10–19 years [18]. Younger children were also eligible because the review specifically addressed both child and adolescent athletes.

Both directions of the association were considered: psychological factors associated with subsequent injury occurrence and mental health or psychosocial outcomes following sports injury.

Studies were excluded if they involved participants older than 19 years without separately reported or independently extractable data for participants aged 19 years or younger; examined non-sport injuries; did not assess an injury-related psychological or psychosocial variable; or were reviews, case reports, editorials, conference abstracts, protocols, or consensus statements.

No restrictions were applied regarding sex, sport type, competitive level, country, or injury type. Only full-text articles published in English or Spanish between 2021 and 2026 were included. The 2021 lower publication-date limit was selected a priori to focus the review on the most recent evidence and on contemporary approaches to the assessment and management of psychological and psychosocial factors in youth sports injury. This restriction was intended to provide an updated synthesis of the rapidly evolving literature rather than a historical overview of the field.

### 2.3. Information Sources and Search Strategy

A systematic literature search was conducted in PubMed, Web of Science, Scopus, and Google Scholar. Searches covered all records available from database inception to 8 July 2026. Search terms related to sports injury, mental health, children, adolescents, and athletes were combined using the Boolean operators “AND” and “OR”. The search strategy prioritized broad mental-health constructs and several commonly reported psychological outcomes rather than exhaustively listing every psychosocial variable subsequently identified during study screening. For Google Scholar, the first 300 results returned by the search were screened in the order provided by the platform. The search was conducted on 8 July 2026. Given the supplementary nature of Google Scholar and the large number of results retrieved, screening was limited to the first 300 records. The search strategy was adapted to the syntax of each database. Reference lists of eligible studies and relevant reviews were also screened to identify additional studies. The complete search strategies are presented in Table 1.

### 2.4. Study Selection

All records identified through the database searches were imported into a reference management program, and duplicate records were removed. Two reviewers independently screened titles and abstracts according to the predefined eligibility criteria. Potentially relevant articles were subsequently assessed in full.

**Figure 1:**
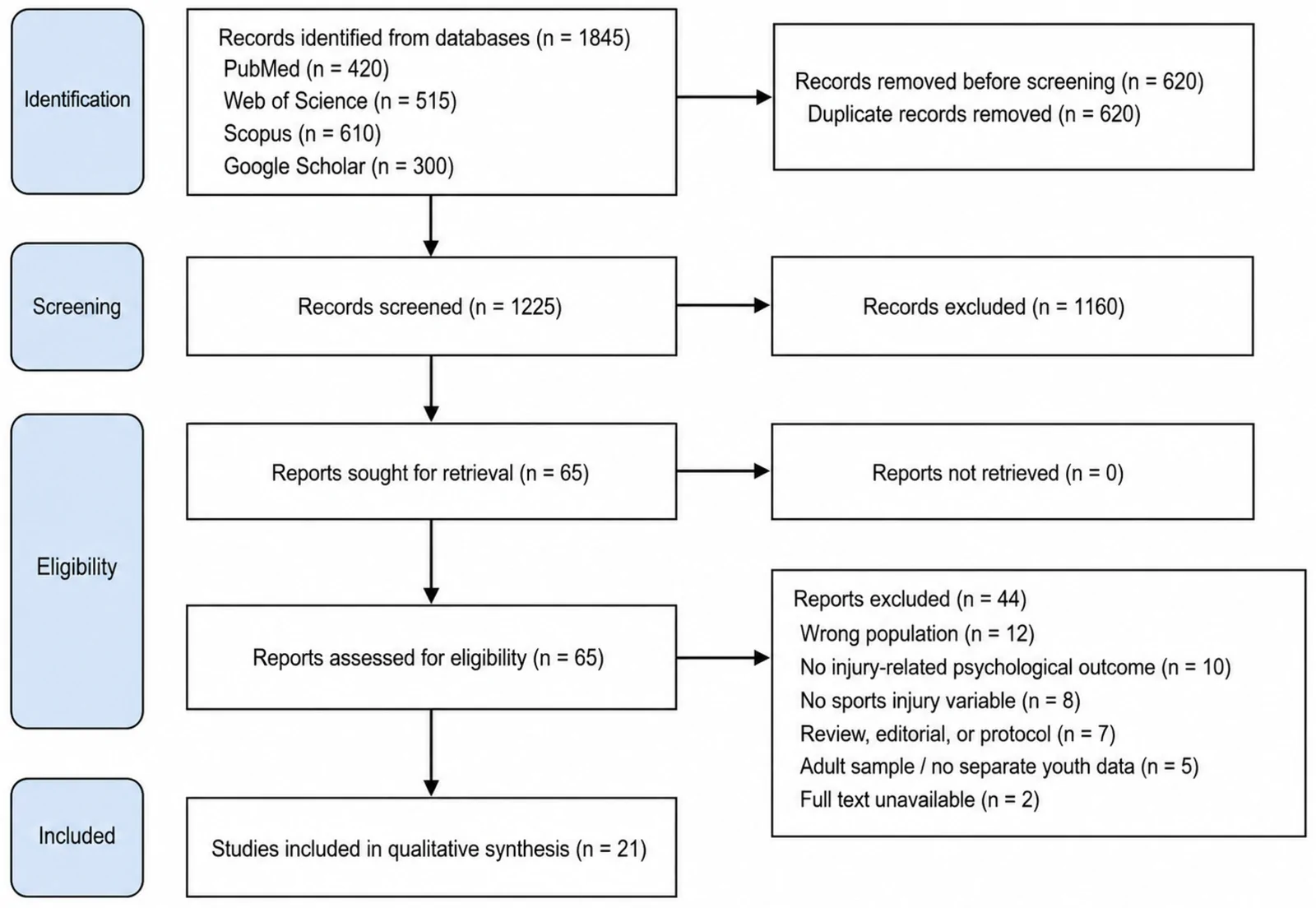
PRISMA 2020 flow diagram of the study selection process. Note. The flow diagram was prepared in accordance with the PRISMA 2020 statement.

Disagreements between reviewers were resolved through discussion, and when necessary, consultation with a third reviewer. Reasons for excluding articles at the full-text stage were recorded. The study selection process is presented in the PRISMA 2020 flow diagram (Figure 1).

The database search identified 1845 records: 420 from PubMed, 515 from Web of Science, 610 from Scopus, and 300 from Google Scholar. After removing 620 duplicate records, 1225 records were screened by title and abstract. Of these, 1160 were excluded. Sixty-five full-text reports were assessed for eligibility, and 44 were excluded because of an ineligible population, absence of an injury-related psychological outcome, absence of a sports-injury variable, publication type, adult samples without separate youth data, or unavailable full text. A total of 21 studies were included in the qualitative synthesis.

### 2.5. Data Extraction

Data were extracted independently by two reviewers using a standardized data-extraction form. The following information was collected from each study: authors and year of publication, country, study design, participant characteristics, sample size, sport and injury type, study objective, psychological or psychosocial variables, assessment instruments, and main findings.

Any discrepancies between reviewers were resolved through discussion, and when necessary, consultation with a third reviewer. When relevant information was unclear or incomplete, the full text and Supplementary Materials were reviewed before a final decision was made.

### 2.6. Risk-of-Bias Assessment

The risk of bias of the included studies was assessed independently by two reviewers using the Joanna Briggs Institute critical appraisal tools appropriate to each study design [19]. Design-specific JBI critical appraisal tools were applied to cohort, analytical cross-sectional, case series, and qualitative studies. For Castellana et al. [20], an exploratory secondary analysis of two randomized controlled trials, the JBI Critical Appraisal Checklist for Randomized Controlled Trials was used, with particular attention to randomization, allocation, follow-up, outcome measurement, statistical analysis, and deviations from the original trial design. Because the mood-symptom subgroup analysis was exploratory and retrospectively defined rather than a prespecified primary analysis of the original trials, the possibility of selective reporting could not be excluded. This limitation contributed to a more cautious interpretation of the intervention finding and to the classification of the study as having some concerns regarding risk of bias.

Each criterion was rated as “yes”, “no”, “unclear”, or “not applicable”. No study was excluded on the basis of the risk-of-bias assessment, and no formal sensitivity analysis or certainty-of-evidence grading was performed. Instead, risk-of-bias judgements were used to guide the narrative interpretation: findings from studies with higher risk of bias were given less interpretative emphasis and were not used as primary support for the review conclusions.

For descriptive purposes, overall risk-of-bias judgements were derived from the pattern of responses across the applicable JBI items. Studies with all or nearly all applicable criteria rated “yes” and no major methodological limitation were classified as low risk of bias; studies with one or more “no” or “unclear” ratings indicating potentially important but not critical limitations were classified as having some concerns; and studies with multiple “no” or “unclear” ratings affecting key domains such as participant selection, exposure or outcome measurement, confounding, follow-up, or statistical analysis were classified as high risk of bias. These categories were used as an interpretative summary and are not formal JBI scoring categories.

Because several studies assessed multiple psychological or psychosocial outcomes using different instruments, outcome-level assessments were also conducted for domains related to outcome measurement and missing data. These assessments considered the appropriateness and validity of the instrument used for each outcome, the consistency of outcome measurement across participants, and the extent and management of missing outcome data. Outcome-specific judgements are reported in Supplementary Table S1, while overall study-level risk-of-bias judgements are summarized in the Results section.

For studies co-authored by members of the present review team, the risk-of-bias assessment was independently verified by a third reviewer who had no authorship involvement in the primary study. Any disagreements regarding item-level assessments, outcome-specific judgements, or overall risk-of-bias classifications were resolved through discussion among the reviewers.

### 2.7. Data Synthesis

Given the substantial heterogeneity in study designs, injury types, psychological and psychosocial outcomes, assessment instruments, and effect measures, statistical pooling was not considered appropriate. The feasibility of quantitative synthesis was also considered for subsets of studies addressing similar injury–outcome combinations. However, even within the more frequently represented areas, such as sport-related concussion and ACL injury, studies differed in design, participant characteristics, outcome definitions, assessment instruments, follow-up periods, and reported effect measures. Consequently, no subset of studies was considered sufficiently homogeneous to support a methodologically appropriate pooled estimate. Findings were therefore synthesized using a structured narrative approach. Studies were first grouped according to the direction of the association examined: (1) psychological or psychosocial factors associated with subsequent injury occurrence, symptom burden, recovery, or reinjury; and (2) mental health or psychosocial outcomes following sports injury. Within these groups, findings were compared according to injury type, psychological outcome, stage of recovery, and study design. When findings were inconsistent or conflicting, differences in study design, participant characteristics, injury definition, outcome measurement, follow-up period, and risk of bias were examined as potential explanations, and divergent findings were retained rather than being reduced to a single overall direction of effect.

Quantitative findings were presented using the effect measures reported in the primary studies where available; effect estimates were not transformed or standardized across studies because of differences in study designs, outcomes, instruments, and analytical approaches. Sample size was not used as a statistical weighting factor, and studies were not formally ranked. Instead, the interpretation considered study design, temporal direction, sample size, measurement approach, and risk of bias, with a more cautious interpretation of findings from small, cross-sectional, retrospective, or higher-risk studies.

Qualitative evidence was synthesized separately by identifying recurrent themes related to emotional responses, athletic identity, social support, communication, rehabilitation experiences, and return-to-sport confidence. These findings were subsequently integrated with the quantitative evidence through narrative comparison, examining whether qualitative themes converged with, complemented, or provided contextual explanations for quantitative associations. Qualitative and quantitative findings were not statistically combined. Individual study characteristics and findings are summarized to facilitate comparison across studies.

## 3. Results

### 3.1. Study Characteristics

A total of 21 studies were included in the qualitative synthesis. The main characteristics of the included studies are presented in Table 2, including publication year, country, study design, sample characteristics, sport or injury type, and study objective.

The studies were published between 2021 and 2026 and were conducted in the United States, Sweden, Spain, Australia, Ireland, and Cuba. The included evidence comprised prospective and retrospective cohort studies, cross-sectional studies, observational recovery studies, clinical cohorts, one case series, one secondary analysis of randomized controlled trials, and qualitative studies.

Study populations included youth athletes with sport-related concussion, anterior cruciate ligament reconstruction, ankle injury, acute and overuse musculoskeletal injuries, previous injury history, and athletes prospectively monitored for injury risk. Psychological and psychosocial variables included anxiety, depressive symptoms, subjective well-being, stress, sleep quality, fatigue, health-related quality of life, kinesiophobia, fear of reinjury, confidence, athletic identity, and psychological readiness to return to sport.

Psychological variables, assessment instruments, main findings, and risk of bias are summarized in Table 3.

### 3.2. Main Findings

The included studies showed substantial heterogeneity in study design, injury type, psychological variables, and timing of assessment. Overall, the evidence addressed two complementary directions of association: psychological and psychosocial factors as potential predictors of sports injury, and mental health or psychosocial outcomes following injury.

Several prospective studies examined psychological factors preceding injury. Lower subjective well-being was associated with greater subsequent injury risk and severity in adolescent elite athletes, while more severe injuries were also associated with poorer well-being [21]. In youth floorball players, higher stress, poorer sleep, and lower well-being were associated with increased injury risk during the following week [28]. Baseline mental well-being and previous mental health difficulties were also examined as predictors of later injury among elite adolescent athletes [37]. Competitive anxiety, particularly worry and concentration disruption, was associated with greater injury vulnerability in youth soccer players [32], while higher cognitive and somatic anxiety predicted injury occurrence, number, and severity in young athletes [39].

Sport-related concussion was one of the most frequently investigated injury types. Shortly after concussion, adolescent athletes reported impairments in anxiety, depression, fatigue, pain interference, physical function, and peer relationships, with most domains improving during recovery and by return to sport [23]. Health-related quality of life also improved progressively after concussion or acute ankle injury, although concussion was associated with greater impairment in school functioning and ankle injury with greater physical limitations [24]. Greater sleep-related symptom severity was associated with persistent symptoms and prolonged concussion recovery [25]. A history of concussion was additionally associated with poorer sleep quality across several domains [33]. Adolescents with a high burden of new-onset mood symptoms showed improved recovery outcomes when prescribed sub-symptom aerobic exercise [20].

Psychological recovery after musculoskeletal injury was addressed particularly in athletes undergoing anterior cruciate ligament reconstruction. Greater psychological readiness was associated with subsequent return to competitive sport [26], whereas higher kinesiophobia and fear of reinjury were associated with poorer readiness to return to sport [31]. Postoperative psychological readiness scores were also associated with later ipsilateral or contralateral reinjury risk in adolescent athletes [38].

Health-related quality of life and broader psychosocial functioning were examined across different sports injuries. Injured female youth volleyball players experienced reductions in total, physical, school, and psychosocial quality of life, particularly following more severe or season-ending injuries [22]. Injury type was associated with different health-related quality-of-life profiles, with concussion linked to greater fatigue and academic difficulties and overuse injuries to poorer mobility [30]. Longitudinal evidence indicated that most quality-of-life domains improved over time, although outcomes varied according to sex, surgical treatment, and discontinuation of sport [36]. Currently injured youth soccer players reported greater anxiety and depressive symptoms and poorer quality of life than uninjured players, with some differences persisting after return to play [40].

Qualitative evidence highlighted the emotional and social consequences of injury. Young elite athletes described frustration, fear of falling behind, and the importance of communication and support during rehabilitation [27]. Injured athletes also reported emotional distress, loss of athletic identity, social pressure to continue participating, reduced confidence, and challenges during return to sport [35].

Other studies examined contextual factors related to mental health and injury. Among youth softball players, previous arm overuse injury was associated with higher depressive symptoms, whereas sport specialization itself was not consistently associated with anxiety or depression [29]. Fatigue and mental health indicators were associated with injury patterns in young rugby players [34]. Across the included studies, the strength and direction of associations varied according to injury type, sport context, assessment method, and study design.

### 3.3. Risk of Bias

Risk of bias varied across the 21 included studies. Nine studies were rated as having a low risk of bias, eleven as having some concerns, and one as having a high risk of bias.

The most common methodological limitations were cross-sectional or retrospective designs, reliance on self-reported injury and psychological data, limited control of potential confounding variables, small or selected samples, and incomplete information regarding follow-up or participant attrition. Several studies also assessed injury history and mental health variables at the same time, limiting conclusions about temporality and causality.

Studies rated as having a low risk of bias generally used prospective or longitudinal designs, clearly defined samples, validated assessment instruments, repeated measurements, and systematic injury surveillance. Studies classified as having some concerns typically provided relevant evidence but were limited by cross-sectional analyses, retrospective clinical data, self-reported outcomes, or insufficient adjustment for confounders. The study rated as having a high risk of bias had greater limitations related to study design, measurement, and control of alternative explanations.

## 4. Discussion

The aim of this systematic review was to synthesize the available evidence on the relationship between sports-related injuries and mental health in child and adolescent athletes aged 19 years or younger. Specifically, it sought to identify the principal mental health and psychosocial outcomes associated with injury, determine whether psychological factors may operate as antecedents or consequences of injury, and describe their role across injury occurrence, rehabilitation, and return to sport. Overall, the findings support a complex, multifactorial, and potentially bidirectional relationship. Lower well-being, competitive anxiety, stress, sleep disturbance, fatigue, kinesiophobia, and reduced psychological readiness were associated with injury occurrence, recovery, or reinjury in some studies. Conversely, injury was associated with anxiety, depressive symptoms, emotional distress, sleep problems, reduced health-related quality of life, disruption of athletic identity, lower confidence, and difficulties returning to sport.

This overall pattern is consistent with the review of reviews by Gil-Caselles et al. [41], which concluded that mental health symptoms may precede sports injury and increase vulnerability, while injury may simultaneously contribute to psychological distress and interfere with recovery. It also accords with the systematic review and meta-analysis by Chow et al. [11], which identified associations in both directions among adolescents and young adults. However, Chow et al. found that the prospective association between poorer mental health and subsequent injury weakened after accounting for potential publication bias. Accordingly, the available evidence more consistently supports psychological consequences following injury than the capacity of psychological variables to predict subsequent injury. The findings should therefore be interpreted as evidence of reciprocal interaction rather than proof of a direct causal cycle.

The prospective studies included in this review provide the strongest evidence regarding psychological factors preceding injury. Lower subjective well-being was associated with greater subsequent injury risk and severity among elite adolescent athletes [21]. Similarly, higher stress, poorer sleep, and lower well-being were associated with increased injury risk during the following week in youth floorball players [28]. Lindman et al. also prospectively examined mental well-being and previous mental health difficulties in relation to subsequent injury [37]. These findings are compatible with the integrated biopsychosocial perspective described in the sport-injury literature, in which injury risk reflects interactions among stress exposure, coping resources, physiological responses, training demands, recovery, and the athlete’s social environment [2,10,15]. Psychological well-being should therefore be understood as one component of a broader and dynamic capacity to cope with sporting and non-sporting demands rather than as an isolated risk factor.

The short assessment intervals used in some prospective studies are particularly informative. Weekly fluctuations in well-being, stress, fatigue, and sleep may capture changes associated with training load, competition, academic pressure, interpersonal difficulties, and insufficient recovery more effectively than a single baseline assessment. This interpretation is consistent with the consensus statement by Tranaeus et al. [10], which conceptualized psychological variables as dynamic factors operating across injury risk, rehabilitation, return to sport, and reinjury. Nevertheless, these measures should not be interpreted as tools for predicting which individual athlete will become injured, because their meaning depends on concurrent factors such as exposure, previous injury, maturation, physical conditioning, sleep, training load, and sport-specific demands.

Competitive anxiety was another relevant factor. Higher competitive anxiety, particularly worry and concentration disruption, was associated with greater injury vulnerability in youth soccer players [32], while cognitive and somatic anxiety were related to injury occurrence, number, and severity among young Cuban athletes [39]. Excessive anxiety may plausibly interfere with attentional control, anticipation, decision-making, and motor coordination, whereas somatic arousal may increase muscle tension and reduce movement efficiency. These mechanisms are consistent with broader psychological models of sports injury [10,16]. However, the included anxiety studies were cross-sectional or correlational, meaning that anxiety may have preceded injury, resulted from previous injury experiences, or reflected other factors such as competitive level and training exposure. These findings therefore support an association but do not establish anxiety as an independent or deterministic cause of injury.

The evidence concerning fatigue and sleep further supports this multifactorial interpretation. Poorer sleep was associated with subsequent injury risk in youth floorball players [28], whereas greater perceived fatigue was related to injury patterns in young rugby players [34]. Sleep loss and accumulated fatigue may influence attention, reaction time, emotional regulation, neuromuscular control, pain perception, and physical recovery. These observations are consistent with pediatric sport literature emphasizing interactions among intensive training, insufficient recovery, overuse injury, and burnout [3,4]. However, the rugby study had a high risk of bias and a small sample, and its findings should therefore be regarded as supportive rather than conclusive.

Findings concerning sport specialization were more nuanced. Zeller et al. [29] found that previous arm overuse injury was associated with higher depressive symptom scores in youth softball players, whereas high specialization itself was not associated with greater anxiety or depression. This suggests that specialization should not automatically be regarded as a direct psychological risk factor. Its effects may depend on the combination of year-round training, excessive volume, reduced autonomy, adult pressure, limited recovery, and previous pain or injury. This interpretation is consistent with Brenner et al. [4] and Brenner et al. [5], who described the physical and psychosocial consequences of specialization as context dependent. The injury experience and the conditions under which specialization occurs may therefore be more relevant to mental health than specialization status alone.

Regarding the consequences of injury, the included evidence indicated impairments across the emotional, physical, social, and academic domains. Injured female volleyball athletes experienced reductions in total, physical, school, and psychosocial quality of life [22]. Youth soccer players with a current injury reported greater anxiety and depressive symptoms and poorer quality of life than uninjured athletes, with some differences persisting after return to play [40]. Verma et al. found that most health-related quality-of-life outcomes improved over time, although trajectories differed according to sex, surgical treatment, and sport discontinuation [36]. These findings are consistent with previous pediatric literature describing sadness, frustration, social isolation, loss of confidence, and uncertainty during injury recovery [1,7,8,9].

The psychological impact of injury may be particularly pronounced during adolescence because sport often provides daily structure, peer belonging, perceived competence, identity, and future aspirations. Temporary removal from sport may therefore involve more than the loss of physical activity; it may disrupt the athlete’s social role and sense of self. Park et al. [13] similarly concluded that stronger athletic identity may increase vulnerability to depressive symptoms following musculoskeletal injury in pediatric athletes. The emotional response to injury is therefore unlikely to be explained solely by tissue damage or recovery duration, but also by the meaning of sport participation and the availability of alternative sources of identity, social connection, and competence.

The qualitative evidence strongly supported this interpretation. Young elite athletes described frustration, fear of falling behind, and the importance of access to care and communication during rehabilitation [27]. Summersby et al. identified emotional distress, identity disruption, social pressure to continue participating despite pain, and reduced confidence during return to sport [35]. These findings are consistent with the qualitative systematic review by Sheehan et al. [12], which showed that adolescent experiences of sports-related pain and injury are shaped by social relationships, expectations, validation, and sporting culture. Psychological responses should therefore not be understood exclusively as individual characteristics, but also as experiences shaped by coaches, parents, peers, clinicians, and sporting organizations.

Sport-related concussion was the most frequently represented injury type. Shortly after concussion, athletes reported impairments in anxiety, depression, fatigue, pain interference, physical function, peer relationships, and other health domains, followed by general improvement during recovery [23]. Health-related quality of life also improved over time after concussion or ankle injury, although concussion was associated with greater disruption of school functioning and ankle injury with greater physical limitation [24]. These injury-specific patterns indicate that psychological and psychosocial consequences are not uniform across conditions. Concussion may particularly disrupt cognitive activity, academic participation, sleep, and symptom tolerance, whereas musculoskeletal injuries may produce more visible restrictions in mobility and physical independence.

Sleep emerged as an important component of concussion recovery. Greater sleep-related symptom severity was associated with persistent symptoms and longer recovery [25], while a history of concussion was associated with poorer sleep duration, latency, disturbance, daytime functioning, and overall sleep quality [33]. These findings are corroborated by the systematic review and meta-analysis by Noordeen et al. [42], which concluded that concussion significantly disrupts sleep quality in adolescents, although variation across studies remains. Sleep disturbance may be both a consequence of concussion and a contributor to fatigue, irritability, anxiety, depressive symptoms, pain sensitivity, and concentration difficulties, supporting its relevance as a recovery domain rather than merely a secondary symptom.

Castellana et al. [20] provide an important intervention-related perspective. Adolescents with a high burden of new-onset mood symptoms had a lower risk of persistent post-concussive symptoms when prescribed subsymptom aerobic exercise. This finding is consistent with randomized evidence showing that individualized subsymptom aerobic exercise can accelerate recovery in adolescents with sport-related concussion [43] and with systematic-review evidence supporting early subsymptom aerobic exercise to reduce symptom burden and the risk of persistent post-concussive symptoms [44]. Castellana et al. extend this literature by suggesting that benefit may be particularly relevant among adolescents with a high burden of new-onset mood symptoms. However, because their study was a secondary analysis of two randomized controlled trials, this subgroup finding should be considered exploratory, and trials specifically designed to test mood-symptom burden as a potential moderator are required.

Psychological readiness and fear of reinjury were particularly relevant after anterior cruciate ligament reconstruction. Greater readiness at six months was associated with return to competitive sport at twelve months [26], whereas greater kinesiophobia was associated with poorer readiness among teenage athletes [31]. McAleese et al. further found that postoperative readiness scores were associated with subsequent ipsilateral or contralateral ACL injury, with sex-specific patterns [38]. These findings reinforce the distinction between physical recovery and psychological readiness: an athlete may satisfy functional criteria while continuing to experience fear, uncertainty, or insufficient confidence.

The present ACL findings agree with the meta-analysis by Xiao et al. [45], in which athletes who returned to sport after ACL reconstruction showed greater psychological readiness, higher self-efficacy, and lower kinesiophobia than those who did not return, despite broadly similar clinical knee-function scores. Readiness scores should nevertheless not be interpreted as isolated clearance thresholds because they may be influenced by recovery stage, sport opportunity, physical status, motivation, and individual risk perception. Sex may also modify psychological recovery. The sex-specific associations reported by McAleese et al. [38] are consistent with the systematic review and meta-analysis by Obradovic et al. [46], which found slightly higher psychological readiness among males than females after ACL reconstruction, although both groups achieved mean values above the proposed return-to-sport threshold. The substantial heterogeneity reported in that meta-analysis indicates that sex alone does not explain readiness; sport type, injury history, rehabilitation context, social expectations, competitive opportunities, and time since surgery may also contribute.

Health-related quality of life was useful for capturing consequences extending beyond symptom burden. The included studies identified changes in physical functioning, fatigue, mobility, school participation, peer relationships, emotional functioning, and perceived health [22,23,24,30,36,40]. Different injuries also produced distinct profiles: concussion was more strongly associated with fatigue and academic difficulties, whereas overuse or acute musculoskeletal injuries were associated with mobility and physical-function limitations [24,30]. These findings highlight the value of multidimensional, age-appropriate patient-reported measures because clinical symptom counts alone may fail to capture relevant social, emotional, and school-related difficulties.

Although most longitudinal findings indicated improvement, group averages should not be interpreted as universal recovery. Some athletes continued to experience anxiety, depressive symptoms, sleep problems, reduced confidence, or poorer quality of life after medical clearance [36,40]. Return to play is therefore not necessarily equivalent to complete psychological recovery. These findings indicate that psychological recovery may follow a different trajectory from physical recovery and may vary across stages of rehabilitation and return to sport.

The risk of bias of the evidence limits the strength of the conclusions. Nine studies were rated as having a low risk of bias, eleven as having some concerns, and one as having a high risk of bias. Cross-sectional and retrospective designs, self-reported injury histories, selected clinical samples, limited adjustment for confounding, and incomplete follow-up were common. These limitations are particularly important when interpreting bidirectionality, because simultaneous measurement of injury history and current psychological symptoms cannot establish whether mental health difficulties preceded injury, emerged afterward, or reflected shared determinants. The strongest evidence therefore comes from prospective studies using repeated psychological assessments and systematic injury surveillance [21,28,37]. Findings from studies with some concerns or high risk of bias were accordingly treated as supportive evidence and were not considered sufficient, in isolation, to underpin the main conclusions.

A further limitation of the evidence base is its concentration on concussion and ACL reconstruction. Although these injuries are clinically important, this imbalance limits generalization to overuse conditions, recurrent injuries, minor injuries, and injuries managed outside specialist services. The studies also varied substantially in age, sex, sport, competitive level, and measurement instruments. These differences may partly explain the inconsistency across findings and further support the use of narrative rather than quantitative synthesis.

The principal contribution of this review is its focus on athletes aged 19 years or younger and its integration of both directions of association across injury occurrence, rehabilitation, and return to sport. In contrast to approaches focused exclusively on psychiatric symptoms after injury, the present synthesis also considered sleep, fatigue, subjective well-being, quality of life, school functioning, identity, confidence, kinesiophobia, and psychological readiness. Although these variables do not necessarily represent mental disorders, they are clinically relevant components of mental health and psychosocial functioning in developing athletes.

Taken together, the findings are consistent with the integrated model of psychological response to sport injury proposed by Wiese-Bjornstal et al. [47], which conceptualizes injury responses as the result of interactions among personal and situational factors, cognitive appraisals, emotional responses, and behavioral outcomes. More recent sport-injury research has further emphasized the dynamic interaction of psychological, physiological, social, and contextual influences across injury risk, rehabilitation, return to sport, and reinjury [10]. However, the associations identified in the present review may also reflect alternative or shared mechanisms, including training exposure, previous injury, physical function, sleep, maturation, rehabilitation stage, and social or sporting context. Psychological factors should therefore not be interpreted as isolated causal determinants, but as components of a broader and dynamic injury process.

## 5. Limitations of the Study

Several limitations should be considered when interpreting the findings of this systematic review. First, the included studies showed substantial heterogeneity in research design, sport type, injury characteristics, participant age, psychological variables, assessment instruments, and timing of measurement. This variability limited direct comparisons across studies and precluded meta-analysis.

Second, many studies used observational, cross-sectional, or retrospective designs, restricting the ability to establish temporal or causal relationships. In several cases, it was not possible to determine whether psychological difficulties preceded injury, emerged as a consequence of injury, or reflected a reciprocal interaction between both processes. Some studies also relied on self-reported injury histories and psychological symptoms, which may have introduced recall and reporting bias.

Risk-of-bias assessments indicated variability in the methodological robustness of the included evidence, with nine studies rated as having a low risk of bias, eleven as having some concerns, and one as having a high risk of bias. The main limitations identified across studies included small or selected samples, limited control of confounding variables, incomplete follow-up, and insufficient reporting of injury exposure, severity, or recurrence.

The evidence was also unevenly distributed across injury types. Sport-related concussion and anterior cruciate ligament reconstruction were more frequently represented than overuse, recurrent, and other musculoskeletal injuries, which may limit the generalizability of the findings across the broader spectrum of youth sports injuries.

Some studies also included narrowly defined samples according to sex, sport, competitive level, or clinical setting, further limiting generalizability to the broader population of child and adolescent athletes. Across the included studies, participant ages ranged from 8 to 19 years, and differences in developmental stage across this broad age range may have influenced psychological responses but were not consistently examined.

The search strategy did not include all potentially relevant psychology-specific databases or all outcome-specific terms, which may have reduced search sensitivity. It also did not explicitly include some youth competitive-category terms (e.g., U18, U-18, under-18, junior, academy) or the British spelling variants ‘paediatric’ or ‘paediatrics’, which may have further limited retrieval. Restricting eligibility to studies published from 2021 onward may also have excluded relevant earlier evidence and should therefore be considered when interpreting the comprehensiveness of the review.

Finally, only full-text articles published in English or Spanish were included. The review protocol was not prospectively registered, although it was subsequently registered retrospectively in PROSPERO (CRD420261459540). Although the review followed the PRISMA 2020 recommendations, these factors may have introduced language, selection, and reporting bias.

## 6. Practical Applications

The findings support incorporating psychological and psychosocial assessment into injury prevention, rehabilitation, and return-to-sport processes in child and adolescent athletes. Screening for anxiety, depressive symptoms, sleep problems, fatigue, fear of reinjury, reduced confidence, impaired quality of life, and poor psychological readiness may help identify athletes who require further assessment or additional support.

Psychological screening should not be used in isolation to predict injury. Its interpretation should consider previous injury, training exposure, physical function, recovery status, sleep, maturation, and sport-specific demands.

Psychological monitoring may be particularly useful when repeated across key stages of recovery, as emotional and psychosocial responses can change from the acute phase through rehabilitation and return to sport. Greater attention may be warranted in athletes with persistent symptoms, prolonged absence from sport, marked identity disruption, poor sleep, high fear of reinjury, or low confidence.

Rehabilitation may benefit from developmentally appropriate psychological support, including psychoeducation, realistic goal setting, graded exposure to activity, confidence-building, emotional support, and strategies to improve communication and adherence. These approaches should be individualized and integrated within the athlete’s broader clinical and sporting context.

Return-to-sport decisions may benefit from considering psychological readiness, fear of reinjury, confidence, and perceived ability to resume sporting demands alongside physical recovery and functional performance. Multidisciplinary collaboration among physicians, physiotherapists, sport psychologists, coaches, families, and athletes may contribute to a more comprehensive recovery process.

## 7. Future Lines of Research

Future research should prioritize prospective and longitudinal designs capable of clarifying the temporal and potentially bidirectional relationship between mental health and sports injury. Psychological and psychosocial variables should be assessed repeatedly from the pre-injury period through rehabilitation, return to sport, and post-return reintegration. Greater methodological standardization is also needed, including consistent definitions of injury, exposure, severity, recurrence, recovery, and return to sport, together with validated and developmentally appropriate measures of psychological outcomes.

Research should also extend beyond concussion and ACL reconstruction to include overuse, recurrent, and other musculoskeletal injuries. Adequately powered studies are needed to determine whether injury-related psychological responses and recovery trajectories differ according to sex, developmental stage, injury severity, sport type, competitive level, training load, specialization, social support, family and coach involvement, and access to healthcare.

Finally, intervention studies are needed to determine whether psychological and multidisciplinary approaches can improve emotional well-being, rehabilitation adherence, confidence, psychological readiness, return-to-sport outcomes, and reinjury risk. Such studies should also identify which athletes are most likely to benefit, which intervention components are effective, and the stage of recovery at which they should be implemented.

## 8. Conclusions

The available evidence indicates that sports injuries and mental health are closely interconnected in child and adolescent athletes. Psychological factors, including lower well-being, competitive anxiety, stress, sleep disturbance, fatigue, kinesiophobia, and reduced psychological readiness, may be associated with injury occurrence, symptom burden, recovery, or reinjury. Conversely, sports injury may negatively affect anxiety, depressive symptoms, sleep, health-related quality of life, confidence, athletic identity, social functioning, sport participation, and readiness to return to sport.

These findings support a potentially bidirectional and biopsychosocial interpretation of sports injury in young athletes. However, the evidence more consistently demonstrates psychological and psychosocial consequences following injury than the capacity of psychological variables to predict subsequent injury, and causal inference remains limited by heterogeneity and the predominantly observational nature of the available studies.

Overall, psychological and psychosocial factors may warrant consideration alongside physical recovery when evaluating injury and return-to-sport processes in young athletes. Further prospective and intervention research is needed to clarify temporal relationships and determine which psychological approaches may improve recovery and reduce reinjury risk.

## Figures and Tables

**Table 1:** Search strategies used across databases.

Database	Search Strategy	Search Period	Records Identified
PubMed	((“sports injury”[Title/Abstract] OR “sport-related injury”[Title/Abstract] OR “athletic injury”[Title/Abstract] OR “Athletic Injuries”[MeSH Terms]) AND (“mental health”[Title/Abstract] OR anxiety[Title/Abstract] OR depression[Title/Abstract] OR “depressive symptoms”[Title/Abstract] OR “psychological distress”[Title/Abstract] OR “fear of reinjury”[Title/Abstract] OR “quality of life”[Title/Abstract]) AND (child*[Title/Abstract] OR adolescen*[Title/Abstract] OR youth[Title/Abstract] OR pediatric[Title/Abstract]) AND (athlete*[Title/Abstract] OR sport*[Title/Abstract]))	March–July 2026	420
Web of Science	TS = ((“sports injury” OR “sport-related injury” OR “athletic injury”) AND (“mental health” OR anxiety OR depression OR “depressive symptoms” OR “psychological distress” OR “fear of reinjury” OR “quality of life”) AND (child* OR adolescen* OR youth OR pediatric) AND (athlete* OR sport*))	March–July 2026	515
Scopus	TITLE-ABS-KEY((“sports injury” OR “sport-related injury” OR “athletic injury”) AND (“mental health” OR anxiety OR depression OR “depressive symptoms” OR “psychological distress” OR “fear of reinjury” OR “quality of life”) AND (child* OR adolescen* OR youth OR pediatric) AND (athlete* OR sport*))	March–July 2026	610
Google Scholar	“sports injury” OR “sport-related injury” “mental health” anxiety depression adolescents youth athletes	March–July 2026	300

Note. Searches covered all records available from database inception to 8 July 2026. Search syntax was adapted to the requirements of each database. Reference lists of eligible studies and relevant reviews were also screened manually to identify additional studies. For Google Scholar, the first 300 results returned by the search were screened in the order provided by the platform on 8 July 2026.

**Table 2:** Characteristics of the included studies.

Authors and Year	Country	Study Design	Sample	Sport/Injury	Objective
von Rosen and Heijne [21]	Sweden	Prospective cohort study	386 elite adolescent athletes aged 15–19 years from seven sports; sex distribution not reported.	Prospectively monitored sports injuries and injury severity over 52 weeks	Explore the association between subjective well-being, sports injury occurrence, and injury severity in adolescent elite athletes.
Watson et al. [22]	United States	Prospective cohort study	2073 female high school volleyball athletes; mean age 15.6 ± 1.1 years.	Time-loss and season-ending sports injuries	Evaluate the impact of in-season injury on quality of life and sleep duration in female youth volleyball athletes.
Williams et al. [23]	United States	Prospective cohort study	70 high school athletes with sport-related concussion; mean age 15.7 years; sex distribution not reported.	Sport-related concussion	Evaluate changes in physical, psychological, and social health domains throughout concussion recovery.
DiSanti et al. [24]	United States	Observational recovery study	85 high school athletes: 46 with concussion and 39 with ankle sprain; sex distribution not reported.	Sport-related concussion or acute ankle injury	Compare health-related quality of life during recovery after concussion or acute ankle injury.
DuPrey et al. [25]	United States	Retrospective cohort study	519 athletes aged 13–18 years with sport-related concussion; sex distribution not reported.	Sport-related concussion	Examine whether sleep-related symptoms are associated with persistent concussion symptoms and prolonged recovery.
Webster and Feller [26]	Australia	Case series	115 adolescent athletes aged ≤17 years after primary ACL reconstruction; sex distribution not reported.	Anterior cruciate ligament reconstruction	Determine whether psychological readiness at 6 months predicts return to competitive sport at 12 months
Ekenros et al. [27]	Sweden	Qualitative interview study	26 elite athletes aged 15–19 years; 14 females and 12 males; participants from 11 individual and team sports.	Previous sports injury requiring rehabilitation	Explore young elite athletes’ experiences of sports-injury rehabilitation, including emotional consequences, access to care, support, and communication
Sonesson et al. [28]	Sweden	Prospective cohort study	471 recreational youth floorball players aged 12–17 years; 142 females; remaining sex distribution not reported.	Prospectively recorded sports injuries during a 26-week season	Investigate whether perceived stress, sleep quality, well-being, sport exposure, and training load were associated with subsequent injury and illness.
Zeller et al. [29]	United States	Cross-sectional survey	1283 female youth softball players aged 12–18 years; mean age 15.1 ± 1.7 years.	Sport specialization behaviors and self-reported arm overuse injury in the previous year	Examine associations between sport specialization behaviors, overuse injury history, anxiety, and depressive symptoms
Verma et al. [30]	United States	Cross-sectional clinical cohort	357 patients aged 8–18 years presenting with injury; mean age 14.2 years; 94% athletes; sex distribution not reported.	Acute injury, overuse injury, and concussion	Compare health-related quality of life according to injury type and athletic participation in injured youth.
Butler et al. [31]	United States	Retrospective cohort study	315 participants aged 13–30 years; teen subgroup: 229 participants aged 13–18 years, analyzed separately and included in this review; sex distribution of the teen subgroup not reported.	Anterior cruciate ligament reconstruction and return-to-sport rehabilitation	Assess the relationship between psychological readiness and kinesiophobia in teenage athletes after ACL reconstruction
Castellana et al. [20]	United States	Exploratory secondary analysis of two randomized controlled trials	198 male and female adolescents aged 13–18 years with sport-related concussion; exact sex distribution not reported.	Sport-related concussion and new-onset mood symptoms	Examine whether early prescribed aerobic exercise reduces the risk of persistent post-concussive symptoms among adolescents with a high burden of new-onset mood symptoms
Sánchez-Ruiz et al. [32]	Spain	Cross-sectional study	322 male youth soccer players aged 10–15 years.	Sports injury history/vulnerability	Analyze the association between competitive anxiety and injury vulnerability.
Sahin et al. [33]	United States	Cross-sectional study	177 adolescent soccer players; mean age 14.61 ± 1.88 years; sex distribution not reported.	Previous concussion, knee injury, or sport-related injury	Examine the association between concussion history and sleep quality in adolescent soccer players while also exploring previous knee injury and other sport-related injury.
Olmedilla-Zafra and Gil-Caselles [34]	Spain	Predictive associative study	23 male rugby players aged 14–17 years from U-16 and U-18 regional teams.	Sports injury occurrence, number, type, and severity	Analyze the relationship between mood, perceived fatigue, and sports injuries according to age category
Summersby et al. [35]	Ireland	Qualitative phenomenological study	17 adolescent athletes aged 15–18 years; mean age 16.2 years; 8 females; remaining sex distribution not reported; participants from five sports.	Traumatic and gradual-onset sports injuries sustained within the previous 2 years	Explore adolescent athletes’ lived experiences of sports injury, including psychological impact, social influences, playing through pain, and confidence when returning to sport
Verma et al. [36]	United States	Longitudinal cohort study	357 patients aged 8–18 years presenting with injury; mean age 14.2 years; 94% athletes; sex distribution not reported.	Acute injuries, overuse injuries, and concussion	Compare health-related quality-of-life outcomes up to 24 months after sport-related injury according to injury type, sex, age, surgery, and sport attrition
Lindman et al. [37]	Sweden	Prospective cohort study	171 elite adolescent athletes aged 16–19 years; sex distribution not reported.	Prospectively registered sports injuries	Evaluate mental well-being and its association with subsequent sports injury risk in elite adolescent athletes
McAleese et al. [38]	Ireland	Cohort study	539 adolescent athletes aged <18 years who underwent primary ACL reconstruction; sex distribution not reported	Primary ACL reconstruction and subsequent ipsilateral or contralateral ACL injury	Examine the longitudinal association between psychological readiness to return to sport and the risk of subsequent ACL injury in adolescent athletes.
Ríos-Garit et al. [39]	Cuba	Cross-sectional correlational study	131 young athletes; mean age 16.49 years; predominantly male; exact sex distribution not reported.	Injury occurrence, frequency, and severity	Examine whether competitive anxiety predicts injury occurrence, number, and severity.
Staresinic et al. [40]	United States	Cross-sectional study	668 elite youth soccer athletes aged 13–19 years; sex distribution not reported.	Current injury, recovered injury, or no recent injury	Examine the association between injury status, anxiety, depressive symptoms, and health-related quality of life in youth soccer players

Note. Sex/gender information is reported within the Sample column according to the information provided in the primary studies.

**Table 3:** Psychological variables, instruments, and main findings.

Authors and Year	Psychological/Psychosocial Variables	Instruments	Main Findings	Overall Risk-of-Bias Judgement
von Rosen and Heijne [21]	Subjective well-being; injury occurrence; injury severity	Weekly injury and well-being questionnaire	Higher well-being predicted lower injury risk (5.6% lower odds per 10-point increase; *p* = 0.036) and lower injury severity (−0.4 points; 95% CI −0.6 to −0.1; *p* = 0.010).	Low risk of bias
Watson et al. [22]	Quality of life; psychosocial functioning; sleep duration	PedsQL and self-reported sleep duration	Injured athletes showed greater declines in total QOL (β = −1.3 ± 0.5; *p* = 0.012), physical function (β = −1.6 ± 0.6; *p* = 0.012), and school function (β = −2.0 ± 0.76; *p* = 0.010).	Low risk of bias
Williams et al. [23]	Anxiety; depression; fatigue; pain interference; physical function; peer relationships	PROMIS Pediatric-25	Physical function, anxiety, depression, fatigue, and pain interference improved significantly during recovery (all *p* < 0.05).	Low risk of bias
DiSanti et al. [24]	Health-related quality of life; physical, emotional, social, and school functioning	Pediatric Quality of Life Inventory (PedsQL)	At 11–29 days, ankle injury was associated with poorer physical function (78.3 ± 19.3 vs. 86.2 ± 15.7; *p* = 0.005), whereas concussion was associated with poorer school function (80.0 ± 20.0 vs. 90.8 ± 12.7; *p* = 0.006).	Some concerns
DuPrey et al. [25]	Sleep difficulty; fatigue; drowsiness; persistent symptoms; recovery duration	SCAT5 symptom scale and clinical records	Each 1-point increase in sleep-symptom severity was associated with a 1.2-fold higher risk of persistent symptoms and 2.4 additional recovery days (both *p* < 0.001).	Some concerns
Webster and Feller [26]	Psychological readiness; emotions; confidence; risk appraisal; return to sport	ACL-RSI scale	ACL-RSI scores improved from 55 to 71 between 6 and 12 months (*p* < 0.001; effect size = 0.98), and 6-month scores predicted return to sport at 12 months (AUC = 0.70, *p* = 0.03).	Some concerns
Ekenros et al. [27]	Frustration; fear of falling behind; support; communication; rehabilitation experiences	Focus-group interviews and content analysis	Qualitative analysis identified four main themes: high-quality rehabilitation, poor healthcare–coach communication, injury-related consequences, and unclear access to rehabilitation; frustration and fear of falling behind were prominent experiences.	Low risk of bias
Sonesson et al. [28]	Stress; sleep quality; well-being; training load; injury risk	Weekly wellness survey and OSTRC questionnaire	Each 1-unit increase in stress, poorer sleep, and poorer well-being increased the odds of injury in the following week by 8% (95% CI 2.0–13.5%), 10% (95% CI 4.2–15.9%), and 8% (95% CI 2.4–13.5%), respectively.	Low risk of bias
Zeller et al. [29]	Anxiety; depressive symptoms; sport specialization; arm overuse injury history	3-point specialization scale, PHQ-9, GAD-7, and self-reported injury history	Athletes with a previous arm overuse injury had higher PHQ-9 scores than those without injury (10.8 ± 0.3 vs. 9.8 ± 0.3; *p* < 0.01). High specialization was not associated with worse anxiety or depression scores.	Some concerns
Verma et al. [30]	Anxiety; depression; fatigue; pain interference; peer relationships; mobility	PROMIS Pediatric-25 and CLASS	Concussed athletes reported greater fatigue (*p* = 0.008), while athletes with overuse injuries had poorer mobility (*p* = 0.005); 90.5% of concussed participants reported worsening grades.	Some concerns
Butler et al. [31]	Psychological readiness; kinesiophobia; fear of reinjury	ACL-RSI and Tampa Scale of Kinesiophobia-11	In the teen subgroup, greater kinesiophobia was moderately associated with lower psychological readiness (*r* = −0.59, *p* < 0.001).	Some concerns
Castellana et al. [20]	New-onset mood symptoms; concussion symptoms; persistent symptoms; recovery	Post-Concussion Symptom Inventory and clinical recovery assessment	In adolescents with high mood-symptom burden, prescribed aerobic exercise was associated with a lower risk of persistent post-concussive symptoms (HR = 0.290, 95% CI 0.123–0.683; *p* = 0.005). This exploratory subgroup finding was interpreted cautiously.	Some concerns
Sánchez-Ruiz et al. [32]	Competitive anxiety; worry; concentration disruption; injury occurrence	Sport Anxiety Scale-2 and injury record	Injury prevalence was 66.5%; two or more injuries increased across age categories (23.6% Alevín, 33.0% Infantil, 39.5% Cadete), and Cadete players showed significant differences in competitive anxiety, including Worry (*p* < 0.001).	Some concerns
Sahin et al. [33]	Sleep duration; sleep latency; sleep disturbance; daytime dysfunction; sleep quality	Pittsburgh Sleep Quality Index and injury-history questionnaire	Athletes with a concussion history had poorer sleep quality (difference = 0.42 ± 0.06; *p* < 0.001) and higher total PSQI scores (difference = 1.91 ± 0.41; *p* < 0.001).	Some concerns
Olmedilla-Zafra and Gil-Caselles [34]	Mood; perceived fatigue; injury occurrence; injury severity	POMS, Brief Fatigue Inventory, and injury survey	Twelve out of 23 players sustained ≥1 injury; U-18 players sustained more injuries than U-16 players (9 vs. 5), and higher fatigue was associated with greater injury tendency.	High risk of bias
Summersby et al. [35]	Emotional distress; identity loss; social influence; confidence; resilience	Semi-structured interviews and reflexive thematic analysis	Four themes emerged: playing through pain/injury, social influences, psychological impact, and confidence during return to sport; 17 adolescents were analyzed (8 females, 9 males).	Low risk of bias
Verma et al. [36]	Anxiety; depression; fatigue; pain; peer relationships; mobility; quality of life	PROMIS Pediatric-25	No significant long-term HRQoL differences were observed between injured athletes and noninjured controls at 12 or 24 months; females showed poorer HRQoL across several domains over follow-up.	Low risk of bias
Lindman et al. [37]	Mental well-being; previous mental health difficulties; subsequent injury	SWEMWBS and prospective injury registration	Athletes sustaining a new injury had lower SWEMWBS scores than injury-free athletes (24.3 ± 3.6 vs. 25.9 ± 3.8; *p* = 0.01); however, mental health variables did not significantly predict new injury in multivariable analysis.	Low risk of bias
McAleese et al. [38]	Psychological readiness; confidence; emotions; return to play; reinjury	ACL-RSI and five-year clinical follow-up	In males, lower 9-month ACL-RSI predicted ipsilateral reinjury (AUC = 0.71, 95% CI 0.65–0.76); contralateral reinjury was associated with higher ACL-RSI in males at 9 months and females at 6 months.	Low risk of bias
Ríos-Garit et al. [39]	Cognitive anxiety; somatic anxiety; self-confidence; injury occurrence, number, and severity	Competitive Anxiety Inventory-2 and medical records	Injured athletes showed greater competitive anxiety (r = 0.31, *p* < 0.001); anxiety was also associated with injury number (ε^2^ = 0.12, *p* = 0.001) and severity (ε^2^ = 0.17, *p* < 0.001).	Some concerns
Staresinic et al. [40]	Anxiety; depression; quality of life; injury and recovery status	GAD-7, PHQ-9, and PedsQL	Currently injured athletes reported greater anxiety (6.6, 95% CI 5.5–7.7), depression (6.9, 95% CI 5.8–8.1), and poorer QOL (73, 95% CI 70–76) than uninjured athletes.	Some concerns

Note. Verma et al. [30] and Verma et al. [36] reported cross-sectional and longitudinal analyses, respectively, from the same underlying cohort. Quantitative results are presented using the effect measures and summary statistics reported in the primary studies; effect estimates were not recalculated or standardized. For qualitative studies, findings are summarized narratively because quantitative effect estimates are not applicable. CI, confidence interval; HR, hazard ratio; OR, odds ratio; β, regression coefficient; *r*, correlation coefficient; AUC, area under the curve; ε^2^, epsilon-squared effect size; *p*, *p*-value.

## Data Availability

The data supporting the findings of this systematic review are available within the article and its Supplementary Materials.

## Supplementary Materials

The following supporting information can be downloaded at: https://www.mdpi.com/article/10.3390/children13081076/s1, Table S1: Outcome-specific risk-of-bias assessment for outcome measurement and missing outcome data.

## Abbreviations

The following abbreviations are used in this manuscript:

ACL	Anterior Cruciate Ligament
JBI	Joanna Briggs Institute
PRISMA	Preferred Reporting Items for Systematic Reviews and Meta-Analyses
PROMIS	Patient-Reported Outcomes Measurement Information System
PedsQL	Pediatric Quality of Life Inventory
PHQ-9	Patient Health Questionnaire-9
GAD-7	Generalized Anxiety Disorder-7
HRQoL	Health-Related Quality of Life
RTS	Return to Sport

## References

[1] Haraldsdottir K., Watson A.M. (2021). Psychosocial Impacts of Sports-related Injuries in Adolescent Athletes. Curr. Sports Med. Rep..

[2] Hoang L.N., Joshi P., Patel D.R., Apple R.W. (2025). The Psychology of Sports Injuries in Children and Adolescents: Psychosocial, Developmental, and Recovery Aspects to Injury. Int. J. Environ. Res. Public Health.

[3] Daley M.M., Reardon C.L. (2024). Mental Health in the Youth Athlete. Clin. Sports Med..

[4] Brenner J.S., Watson A., Council on Sports Medicine and Fitness (2024). Overuse Injuries, Overtraining, and Burnout in Young Athletes. Pediatrics.

[5] Brenner J.S., LaBotz M., Sugimoto D., Stracciolini A. (2019). The Psychosocial Implications of Sport Specialization in Pediatric Athletes. J. Athl. Train..

[6] Pluhar E., McCracken C., Griffith K.L., Christino M.A., Sugimoto D., Meehan W.P. (2019). Team Sport Athletes May Be Less Likely to Suffer Anxiety or Depression than Individual Sport Athletes. J. Sports Sci. Med..

[7] Jeong L., Li D. (2024). Psychological Well-Being From Sports Injuries in Adolescence: A Narrative Review. Cureus.

[8] Haugen E. (2022). Athlete Mental Health & Psychological Impact of Sport Injury. Oper. Tech. Sports Med..

[9] Xu E., Greif D.N., Castle P., Lander S. (2025). Pediatric Sports: The Mental Health and Psychological Impact of Sport and Injury. J. Clin. Med..

[10] Tranaeus U., Gledhill A., Johnson U., Podlog L., Wadey R., Wiese-Bjornstal D.M., Ivarsson A. (2024). 50 Years of Research on the Psychology of Sport Injury: A Consensus Statement. Sports Med..

[11] Chow A.R.W., Zaneva M., Rashid L., Wheatley C., Coussios C., Hepach R., Bowes L. (2026). Bidirectional Relationship Between Mental Health and Sports Injury in Adolescents: A Systematic Review and Meta-analysis. Sports Med..

[12] Sheehan N., Summersby R., Bleakley C., Caulfield B., Matthews M., Klempel N., Holden S. (2024). Adolescents’ Experience with Sports-related Pain and Injury: A Systematic Review of Qualitative Research. Phys. Ther. Sport.

[13] Park A.L., Furie K., Wong S.E. (2023). Stronger Athlete Identity Is a Risk Factor for More Severe Depressive Symptoms After Musculoskeletal Injury in Pediatric Athletes: A Systematic Review. Curr. Rev. Musculoskelet. Med..

[14] Hsu C.J., Meierbachtol A., George S.Z., Chmielewski T.L. (2017). Fear of Reinjury in Athletes. Sports Health.

[15] Palisch A.R., Merritt L.S. (2018). Depressive Symptoms in the Young Athlete after Injury: Recommendations for Research. J. Pediatr. Health Care.

[16] Zambak Ö., Toktas S. (2025). Utilization Sports Psychology to Prevent Sports Injury: A Comprehensive Systematic Review. Int. J. Recreat. Sports Sci..

[17] Page M.J., McKenzie J.E., Bossuyt P.M., Boutron I., Hoffmann T.C., Mulrow C.D., Shamseer L., Tetzlaff J.M., Akl E.A., Brennan S.E. (2021). The PRISMA 2020 statement: An updated guideline for reporting systematic reviews. BMJ.

[18] World Health Organization Adolescent Health. https://www.who.int/health-topics/adolescent-health.

[19] Joanna Briggs Institute (2026). JBI Critical Appraisal Tools.

[20] Castellana M.C., Burnett G.J., Gasper A., Nazir M.S.Z., Leddy J.J., Master C.L., Mannix R.C., Meehan W.P., Willer B.S., Haider M.N. (2025). Adolescents with a high burden of new-onset mood symptoms after sport-related concussion benefit from prescribed aerobic exercise: A secondary analysis of two randomized controlled trials. Clin. J. Sport Med..

[21] von Rosen P., Heijne A. (2021). Subjective well-being is associated with injury risk in adolescent elite athletes. Physiother. Theory Pract..

[22] Watson A., Biese K., Kliethermes S.A., Post E., Brooks M.A., Lang P.J., Bell D.R., Haraldsdottir K., McGuine T. (2021). Impact of in-season injury on quality of life and sleep duration in female youth volleyball athletes: A prospective study of 2073 players. Br. J. Sports Med..

[23] Williams R.M., Johnson R.S., Snyder Valier A.R., Bay R.C., Valovich McLeod T.C. (2021). Evaluating multiple domains of health in high school athletes with sport-related concussion. J. Sport Rehabil..

[24] DiSanti J.S., Marshall A.N., Valier A.R.S., McLeod T.C.V. (2022). High school athletes’ health-related quality of life across recovery after sport-related concussion or acute ankle injury: A report from the Athletic Training Practice-Based Research Network. Orthop. J. Sports Med..

[25] DuPrey K.M., Char A.S., Loose S.R., Suffredini M.V., Walpole K., Cronholm P.F. (2022). Effect of sleep-related symptoms on recovery from a sport-related concussion. Orthop. J. Sports Med..

[26] Webster K.E., Feller J.A. (2022). Psychological Readiness to Return to Sport After Anterior Cruciate Ligament Reconstruction in the Adolescent Athlete. J. Athl. Train..

[27] Ekenros L., Fridén C., von Rosen P. (2023). Experiences of rehabilitation in young elite athletes: An interview study. BMJ Open Sport Exerc. Med..

[28] Sonesson S., Dahlström Ö., Panagodage Perera N.K., Hägglund M. (2023). Risk factors for injury and illness in youth floorball players—A prospective cohort study. Phys. Ther. Sport.

[29] Zeller A.M., Lear A., Post E., McNulty S., Bentley B. (2024). A national survey on the relationship of youth sport specialization behaviors to self-reported anxiety and depression in youth softball players. Sports Health.

[30] Verma R., DeMaio E., Render A., Wild J., Hunt D., Cato S., Shenvi N., LaBella C., Stracciolini A., Jayanthi N. (2024). The effects of injury type on health-related quality of life in youth athletes: A cross-sectional analysis. Clin. J. Sport Med..

[31] Butler L., Baez S., Walker C., Roman D., Douthit T., Kuenze C., Ulman S., ARROW (2025). The relationship between psychological readiness to return to sport and kinesiophobia in teens and young adults after anterior cruciate ligament reconstruction. Front. Psychol..

[32] Sánchez-Ruiz R., Gil-Caselles L., García-Naveira A., Arbinaga F., Ruiz-Barquín R., Olmedilla-Zafra A. (2025). Competitive anxiety, sports injury, and playing category in youth soccer players. Children.

[33] Sahin S., Erdman A.L., Loewen A., Miller S.M., Jones J.C., Chung J.S., Janosky J., Ulman S. (2025). Concussion history is associated with poor sleep quality in adolescent athletes: A cross-sectional study. J. Clin. Sleep Med..

[34] Olmedilla-Zafra A., Gil-Caselles L. (2025). Fatiga, salud mental y lesiones en jugadores jóvenes de rugby. Arch. Med. Deporte.

[35] Summersby R., Sheehan N., Bleakley C., Caulfield B., Matthews M., Klempel N., Holden S. (2025). “I was devastated… sport is like an addiction nearly”: A qualitative study examining young athletes’ experiences with injury in sport. BMJ Open Sport Exerc. Med..

[36] Verma R., Render A., Ramirez M., Shenvi N., Easley K.A., LaBella C., Stracciolini A., Jayanthi N. (2025). Long-term health-related quality of life after sport-related injury in youth athletes: A longitudinal cohort study. Orthop. J. Sports Med..

[37] Lindman I., Mohammadzadijaran E., Baranto A., Abrahamson J. (2026). Mental health and its association with injury risk in elite adolescent athletes—A prospective cohort study. BMC Sports Sci. Med. Rehabil..

[38] McAleese T., Mactier L., Bryan K., King E., Moran K.A., Jackson M., Withers D., Moran R., Devitt B.M. (2026). Postoperative anterior cruciate ligament–return to sport after injury scores predict reinjury in adolescent athletes. Orthop. J. Sports Med..

[39] Ríos-Garit J., Pérez-Surita Y., Gómez-Espejo V., Reyes-Bossio M., Tutte-Vallarino V. (2026). Competitive anxiety as a predictor of the occurrence, quantity, and severity of injuries in young Cuban athletes. Int. J. Environ. Res. Public Health.

[40] Staresinic I., Haraldsdottir K., Sanfilippo-Nackers J., Gupta S., Steiner Q., Anderson S., Watson A. (2026). Impact of injury on anxiety, depression, and quality of life in youth soccer players. Clin. J. Sport Med..

[41] Gil-Caselles L., Ruiz-Barquín R., Giménez-Egido J.M., Olmedilla-Zafra A. (2024). Bidirectional relationship between mental health and sport injuries: A review of reviews. Apunt. Sports Med..

[42] Noordeen S., Wang P., Strazda A.E., Arany E.S., Ergisi M., Yeo L.J., Popovic R., Mahendran A., Khawaja M., Grover K. (2025). Concussion disrupts sleep in adolescents: A systematic review and meta-analysis. Clocks Sleep.

[43] Leddy J.J., Haider M.N., Ellis M.J., Mannix R., Darling S.R., Freitas M.S., Suffoletto H.N., Leiter J., Cordingley D.M., Willer B.S. (2019). Early subthreshold aerobic exercise for sport-related concussion: A randomized clinical trial. JAMA Pediatr..

[44] Leddy J.J., Burma J.S., Toomey C.M., Hayden A., Davis G.A., Babl F.E., Gagnon I., Giza C.C., Kurowski B.G., Silverberg N.D. (2023). Rest and exercise early after sport-related concussion: A systematic review and meta-analysis. Br. J. Sports Med..

[45] Xiao M., van Niekerk M., Trivedi N.N., Hwang C.E., Sherman S.L., Safran M.R., Abrams G.D. (2023). Patients who return to sport after primary anterior cruciate ligament reconstruction have significantly higher psychological readiness: A systematic review and meta-analysis of 3744 patients. Am. J. Sports Med..

[46] Obradovic A., Manojlovic M., Rajcic A., Jankovic S., Andric N., Ralic V., Zlicic T., Aleksic B., Ninkovic S., Veraksa A. (2024). Males have higher psychological readiness to return to sports than females after anterior cruciate ligament reconstruction: A systematic review and meta-analysis. BMJ Open Sport Exerc. Med..

[47] Wiese-Bjornstal D.M., Smith A.M., Shaffer S.M., Morrey M.A. (1998). An Integrated Model of Response to Sport Injury: Psychological and Sociological Dynamics. J. Appl. Sport Psychol..

