# Supplementary material for: Sports Injuries and Mental Health in Child and Adolescent Athletes: A Systematic Review
Source: Children (Basel). 2026 Aug 14;13(8):1076. doi: 10.3390/children13081076 (PMC13511745; doi:10.3390/children13081076)
Supplement: Supplementary file 1 [file children-13-01076-s001.zip › children-4487437-supplementary.pdf]

Supplementary Table S1. Outcome-specific risk-of-bias assessment for outcome measurement and missing outcome data.

Study	Outcome(s)	Instrument	Outcome measurement	Missing outcome data
Castellana et al. [20]	Mood-symptom burden; concussion symptoms; persistent symptoms; recovery	PCSI/PCSS symptom items; clinical recovery assessment	Unclear	Yes
von Rosen and Heijne [21]	Subjective well-being; injury occurrence/severity	Weekly injury and well-being questionnaire	Yes	Unclear
Watson et al. [22]	Total QOL; physical, social, school, emotional and psychosocial functioning; sleep duration	PedsQL; self-reported sleep duration	Yes	Yes
Williams et al. [23]	Anxiety; depression; fatigue; pain interference; physical function; peer relationships	PROMIS Pediatric-25	Yes	Unclear
DiSanti et al. [24]	Global HRQoL; physical, emotional, social and school functioning	PedsQL	Yes	Yes
DuPrey et al. [25]	Sleep difficulty; fatigue; drowsiness; persistent symptoms; recovery duration	SCAT5 symptom scale; clinical follow-up	Yes	Yes
Webster and Feller [26]	Psychological readiness; confidence; emotions; return to sport	ACL-RSI	Yes	Yes
Ekenros et al. [27]	Rehabilitation experiences; emotional and psychosocial consequences; communication and access to care	Focus-group interviews; qualitative content analysis	Yes	N/A
Sonesson et al. [28]	Stress; sleep quality; well-being	Weekly 0–10 self-report ratings	Unclear	Unclear
Zeller et al. [29]	Anxiety; depressive symptoms	GAD-7; PHQ-9	Yes	Yes
Verma et al. [30]	HRQoL: mobility, fatigue, anxiety, depressive symptoms, peer relationships, pain interference	PROMIS Pediatric Profile	Yes	Unclear
Butler et al. [31]	Psychological readiness; kinesiophobia	ACL-RSI; TSK-11	Yes	Yes
Sánchez-Ruiz et al. [32]	Competitive anxiety; somatic anxiety; worry; concentration disruption; injury occurrence	SAS-2; self-reported injury questionnaire	Yes	Unclear
Sahin et al. [33]	Sleep quality; sleep duration; latency; disturbances; daytime dysfunction	Pittsburgh Sleep Quality Index (PSQI)	Yes	Yes
Olmedilla-Zafra and Gil-Caselles [34]	Mood; perceived fatigue; injury occurrence/severity	POMS; Brief Fatigue Inventory; ad hoc injury survey	Unclear	Unclear
Summersby et al. [35]	Psychological impact; identity; social influences; confidence; return-to-sport experiences	Semi-structured interviews; reflexive thematic analysis	Yes	N/A

Verma et al. [36]	HRQoL: anxiety, depression, fatigue, pain, peer relationships, mobility, quality of life	PROMIS Pediatric-25	Yes	Unclear
Lindman et al. [37]	Mental well-being; previous mental health difficulties; subsequent injury	SWEMWBS; additional self-report mental-health item; prospective injury registration	Yes	Unclear
McAleese et al. [38]	Psychological readiness; return to play; ipsilateral and contralateral reinjury	ACL-RSI; 5-year clinical follow-up	Yes	Yes
Ríos-Garit et al. [39]	Cognitive anxiety; somatic anxiety; self-confidence; injury occurrence, number, and severity	CSAI-2/competitive anxiety measure; medical injury records	Yes	Unclear
Staresinic et al. [40]	Anxiety; depression; HRQoL	GAD-7; PHQ-9; PedsQL	Yes	Yes

Note. Outcome-specific assessments were restricted to domains related to outcome measurement and missing outcome data. "Yes" indicates that the outcome was measured using an appropriate method and/or that missing outcome data were adequately addressed or clearly accounted for; "No" indicates a clear methodological limitation; "Unclear" indicates insufficient information in the primary report to support a definitive judgement; N/A, not applicable.
